# Comparison of Canine Behaviour Scored Using a Shelter Behaviour Assessment and an Owner Completed Questionnaire, C-BARQ

**DOI:** 10.3390/ani10101797

**Published:** 2020-10-03

**Authors:** Liam Clay, Mandy B A Paterson, Pauleen Bennett, Gaille Perry, Clive C J Phillips

**Affiliations:** 1 Centre for Animal Welfare and Ethics, University of Queensland, Gatton, Queensland 4343, Australia; mpaterson@rspcaqld.org.au (M.B.A.P.); clive.phillips58@outlook.com (C.C.J.P.); 2 Royal Society for the Prevention of Cruelty to Animals Queensland, Brisbane, Queensland 4076, Australia; 3 School of Psychology and Public Health, La Trobe University, Bendigo, Victoria 3552, Australia; pauleen.bennett@latrobe.edu.au; 4 Delta Society, Summer Hill, New South Wales 2130, Australia; perrygaille@gmail.com

**Keywords:** dog behaviour, behaviour problems, behaviour assessment, canines, shelters, predict, home behaviour

## Abstract

**Simple Summary:**

In shelters, it is usual to conduct a standardised behaviour assessment to identify adoption suitability. The information gathered from the assessment is used to identify the behaviour of the dogs, suitability for adoption and to help to match the dog to an ideal home environment. We investigated if the dogs’ behaviour in the home as reported by owners was reflected in the Royal Society for the Prevention of Cruelty to Animals (RSPCA) Queensland behaviour assessment, conducted on the same dogs during a visit to the shelter. A total of 107 owners and their dogs aged 1–10 years were assessed in-home, by the owners, and in the shelter, by a researcher. The owners completed a questionnaire (Canine Behavioural Assessment and Research Questionnaire (C-BARQ)) prior to the standardised behavioural assessment conducted at the RSPCA Queensland. Regression analysis identified positive correlations between the two for fear, arousal, friendliness and anxiousness, identified in in-home behaviour and the behaviour assessment. This research therefore allowed a greater understanding of current canine behaviour assessment protocols used at the RSPCA Queensland in regard to the predictability of behaviour, behavioural problems and the efficiency and effectiveness of testing procedures.

**Abstract:**

In shelters, it is usual to conduct a standardised behaviour assessment to identify adoption suitability. The information gathered from the assessment is used to identify the behaviour of the dogs, its suitability for adoption and to match the dog with an ideal home environment. However, numerous studies have demonstrated a lack of predictability in terms of the post-adoption behaviour in these assessments. We investigated if the owners’ perception of dogs’ behaviour in the home was reflected in the RSPCA Queensland behaviour assessment, conducted on the same dogs during a visit to the shelter. A total of 107 owners and their dogs aged 1–10 years were assessed in-home and in the shelter. The owners of the dogs completed a questionnaire (the Canine Behavioural Assessment and Research Questionnaire (C-BARQ) survey) 1–2 weeks before bringing their dog to the shelter for the standardised behavioural assessment conducted at the RSPCA Queensland. An ordinal logistic regression analysis identified positive correlations for fear, arousal, friendliness and anxiousness, identified in in-home behaviour and the behaviour assessment. Furthermore, the behaviours of friendliness, fearfulness, arousal, anxiousness, and aggression were positively predictive between home behaviour and tests in the behaviour assessment. This research therefore led to a greater understanding of current canine behaviour assessment protocols used at the RSPCA Queensland in regard to the predictability of behaviour, behavioural problems and the efficiency, effectiveness and predictability of current behaviour testing procedures.

## 1. Introduction

The Royal Society for the Prevention of Cruelty to Animals (RSPCA) Australia accepted 33,863 dogs to its shelters during the period 2018–2019 [1]. Sources of admitted dogs in Queensland include councils, owner surrenders, humane officer admission (employees of the RSPCA with investigative powers under the Queensland Animal Care and Protection Act 2001) and euthanasia requests [2], with age at admission being variable, but with over 74% adult dogs. Dogs are surrendered for numerous reasons: human-related (unwanted, changed circumstances, financial, owner’s health, and ex-commercial/racing), or dog-related (medical and behavioural problems) [3]. After surrender, dogs are housed in the shelter until their suitability for adoption is determined, and if suitable, adopted.

The procedures used to identify dogs suitable for adoption include a medical check, behavioural assessment, in-kennel monitoring, and monitoring by shelter staff when interacting with the dog. Behavioural assessments are the preferred method in many shelters to give an overview of the dog’s behaviour for potential adopters [4,5]. They assess the dog’s reactions to diverse novel stimuli typical of everyday life situations and their ability to cope in challenging situations [6], usually 3–5 days after entering the shelter [5].

The testing procedures have a risk of both false positives and negatives [7,8], that is, running the risk of falsely identifying a behavioural problem that does not exist or deeming a dog suitable for adoption when it is not. These problems may arise due to the stress experienced by the dog from living in the shelter [9], and because certain behaviours are multifactorial and a test carried out at a single point in time may not be able to accurately capture this behaviour. Few studies have evaluated the effect of the timing of behaviour assessments, for example immediately on shelter admission [10].

Measurements used in the assessments need to be appropriate and meaningful, providing both quantitative and qualitative data [11]. Qualitative measurements include history-taking measures, which provide a reflection of previous home environment and behaviour. Current procedures used by RSPCA Queensland are primarily quantitative measures, which are in line with the behaviour assessments reported in the literature that use a direct measure of behaviour by observing the dog’s response to several testing procedures [4,12,13,14,15,16,17]. Other measures focus on the assessment of behaviours in everyday situations, using a questionnaire for the dog’s owner to complete [18,19,20,21]. A widely used questionnaire is the Canine Behavioural Assessment and Research Questionnaire (C-BARQ), which includes items focusing on behaviour associated with aggression, fear and anxiety, trainability, excitability, separation, attachment, attention-seeking, and chasing [18]. It has been extensively evaluated and used to validate quantitative behaviour assessments focusing on areas of behaviour issues and service dogs [22,23,24,25,26].

In order to further investigate the accuracy with which behaviour assessments used in shelters identify behaviours exhibited elsewhere, this study adopted a novel approach to help to determine whether previous home behaviours are accurately reflected in these shelter assessments. The study asked owners to complete a validated questionnaire (C-BARQ) about their dog’s behaviour and then to bring the dog into a shelter where the dog underwent the standardised behaviour assessment. The aim of this study was to determine if the dogs’ behaviour in the home was reflected in the RSPCA Queensland behaviour assessment, conducted on the same dogs during a visit to the shelter.

## 2. Materials and Methods

### 2.1. Ethics

This study was conducted with the approval of the University of Queensland’s Human and Animal Ethics Committees (approval numbers 2018001353 and SVS/290/18, respectively). The study complies with provisions contained in Australia’s National Statement on Ethical Conduct in Human Research and with Queensland regulations governing experimentation on humans.

### 2.2. Subjects

Companion dog owners from the general public (n: 107) were invited via social media to participate in this study. The RSPCA and the University of Queensland media outlets were used to attract participants. Participants had to have owned their current dog for at least 6 months, be over the age of 18 years and willing to complete a questionnaire and bring their dog into the shelter to undergo a non-invasive behaviour assessment. Participants received an information sheet, and if willing to have their dog participate in the study, they signed a consent form outlining that the testing would be used for research purposes. Each participating dog was allocated a number which was used to tie the C-BARQ and assessments to the same dog. Apart from the consent form, all information was non-identifiable and most of the questions focused on information about the dog, not the owner. Owners of dogs had to complete and submit the C-BARQ questionnaire before an appointment was made for the shelter assessment. C-BARQ focuses on the dog’s interactions in numerous situations. The shelter assessment used was the standardised assessment used on all in-coming dogs.

#### Dogs

Dogs were required to be older than 6 months and younger than 13 years of age. Any breed was allowed in the study. Dogs were also required to have no medical conditions nor be on any medication that had the potential to influence behaviour. Dogs previously adopted from shelters were allowed in the study and were initially categorised separately to identify any variability. However, there were no differences between groups, therefore, separate categories were dropped. All dogs were required to be with the owners for at least 6 months.

### 2.3. Behaviour Assessment

The dogs were brought into the shelter by their owner for the formal behaviour assessment. It was conducted in a room (4.5 m × 4.7 m) in a separate building, approximately 50 m from the shelter offices and kennels to minimise disturbance. The dogs were initially left in the room by themselves for 15 min to allow them to acclimatise to the room while the researcher watched their behaviour from the next room via a video link (4× Go pro Hero 4 Silver positioned an equal distance apart). The owner waited in an adjoining area for the period of acclimatisation and assessment.

The behavioural assessment used in this study was the standard assessment used by the RSPCA Queensland for shelter dogs. The assessments were conducted, recorded and scored by the lead researcher (LC), who was formally trained in the assessment regimen. Reviewed behaviours included room exploration, leash manners, sociability, tolerance, play behaviour with toys, the response to unusual/unpredictable stimuli, possessive behaviours, toddler and stranger interaction, time alone and social interactions with other dogs [27] (Appendix A). In each test, the dog’s behaviours were scored for friendliness, socialisation, fearfulness, arousal and aggressiveness. The assessment comprised nine different tests performed over a 15 min period. The equipment used was in line with the RSPCA Queensland’s protocol and included a 1.8 m leash, a tennis ball, a plush squeaky toy, rope, plastic hand on an extend pole, bowl, raw hide or bone, and the combination of wet and dry dog food. The details of the RSPCA Queensland assessment tests can be found in Clay et al. [27]. All the tests were recorded by video (Go Pro Hero 4, Model: HERO4 Black, Manufacture: Hong Kong, China) and reviewed later.

### 2.4. Owner Questionnaire, C-BARQ

Owners rated the behaviour of their dog at home based on behavioural interactions in relation to attachment or attention seeking, sociability, touch sensitivity, excitability, chasing, fear, aggression, and separation-related behaviours. The owners’ information on their dog’s behaviour was categorised into predetermined behavioural categories on a score of 0–4 (Appendix B). The C-BARQ questionnaire used had the 102 question format [24] and was scored on a scale between 0 and 4 (aggression: 0, none—4, serious, separately scored for stranger-, owner-, dog and familiar dog-directed aggression; fear: 0, no fear or anxiety—4, extreme fear, both stranger, non-social and dog fear; separation-related problems: from 0, never, to 4, always; attachment/attention-seeking: from 0, never, to 4, always; touch sensitivity: from 0, never, to 4, always; excitability: from 0, calm, to 4, extremely excitable; chasing, energy, and trainability: from 0, never, to 4, always).

### 2.5. Behaviour Scoring

The formal behaviour assessments were scored for dog behaviour during all tests, as described in Clay et al. [27]. The ethogram comprised 48 behaviours, determined following the preliminary observation of dogs during the formal behaviour assessment, classified as either long duration behaviours (for which the duration was recorded) or events (for which the number of occurrences was recorded). The behaviours focused on eight components: activities of the mouth, body, tail position, tail movement, ears, eyes, position in room, and movement (Table 1). The descriptions of each behaviour were presented in a previous study [27]. Behaviour recording was assisted by coding software BORIS [28], which recorded the frequency and duration of each behaviour using continuous input from the coder. Two behaviour variables with no or only one occurrence were discarded: squint and whale eyes. From the coded behaviours, using similar principles to our previous articles [27,29], the proportion of the time and frequency of the five behavioural categories (anxiety, fear, friendliness, arousal, aggression) were derived. The descriptions of each behaviour are presented in Table 1 and their connection to behavioural categories (anxiety, fear, friendliness, arousal, aggression) in Table 2 are based off the literature described in a previous article (27).

### 2.6. Statistical Analysis

Statistical analysis was conducted using Minitab 18. Behaviours were analysed as the percentage of the total observation time (long duration behaviours) or the percentage of the frequency of occurrence (events) during the overall behaviour assessment and within the individual tests. The C-BARQ questionnaire has predetermined categories that were calculated after the 102 questions were complete. Descriptive analysis was used for behaviour in assessments.

Spearman’s rank order correlations were computed between C-BARQ and the formal behaviour assessment variables. As comparisons with 79 other behaviours were made for each behaviour in each test of the behaviour assessment, results were corrected for false discovery using the Benjamini–Hochberg procedure [30]. The Bonferroni correction was rejected as it assumes the independence of the individual tests. The Benjamini–Hochberg procedure ranks the *p* values for each test and compares the *p* values to critical values [(rank/no. tests) × false discovery rate (selected as 0.20 as recommended by McDonald [30]). All *p* values up to the critical one were considered to indicate a significant difference [30].

Ordinal logistic regression was used to compare the temperament/behavioural information from owner-reported temperament/behaviour with derived behaviours from the shelter assessment, both overall and within the different tests. The Benjamini–Hochberg was used to correct for false discovery as with Spearman rank correlations.

## 3. Results

### 3.1. Descriptive Statistics

The sample included 107 companion dogs (males: 52, females 57, desexed: 103, intact: 6) who were over the age of 6 months and under 13 years (mean: 5 years 3 months). Sources of the dogs included: shelters (44.9%), breeders (23.8%), other (online, private sales, or did not disclose) (11.9%), neighbour, friend, or relative (10.1%), and under 5% were from pet stores or were stray dogs.

A variety of breeds were included in the study, determined by the C-BARQ questionnaire completed by the owners; mixed breeds (19.3%), Border collie (10.1%), Kelpie (8.3%), Staffordshire bull terrier (8.3%), German shepherd (5.5%), Australian cattle dog (3.7%), and Rottweiler (3.7%). All other breeds represented less than 3% of the population of dogs. Mean weight of the dogs was 21.8 ± 1.06 kg.

With respect to the household environment, 64.2% had other dogs in the household; 35.8% were single dog homes. Of the total population, 69.7% of the households had no children and 30.28% had children living in the home. With regard to the living arrangements for the dogs, 80.7% were classified as inside/outside, 12.8% were only inside, 4.6% were only outside and 1.8% had no classification.

### 3.2. Owner Questionnaire

All owners completed the C-BARQ questionnaire (107 participants). Many owners indicated that their dogs displayed no signs of fear (score 0) in situations with other unknown dogs (46%), strangers (68%) and non-social interactions (56%), with the second highest occurrence being the dog displaying minimal signs of fear (score 1) in the above situations (Appendix C). When owners did report that some fear was displayed, it was most likely to be dog directed, then non-social and least likely to be stranger directed.

It was mostly reported that little aggression was observed. In particular, owner-directed aggression was very rare, only 5% of owners reported this, and stranger-directed aggression was also quite rare, with only 28% of owners reporting this, and mostly at low levels. However, dog-related aggression (unfamiliar dogs) was relatively common, reported by 60% of owners, but less towards familiar dogs (34% of owners). Separation-related behaviours were even less common, reported by 23% of owners, but attention-seeking, chasing, excitable and energetic behaviours were relatively common, with most owners reporting some occurrence. Touch sensitivity was less common, with most owners reporting that it was never or seldom seen. Dogs were reported to be trainable most of the time, but never always.

### 3.3. Formal Behaviour Assessment

In the overall formal behaviour assessment, dogs spent 41.2% of their time in friendly behaviours, 28.4% displaying fear, 14.3% in a state of high arousal, 13.5% displaying anxiousness, and 2.5% in aggression. Considering the frequency of the behaviours, there was a mean of 37.6% incidents of friendly behaviours, 30.3% incidents of fear-related behaviours, 15.4% incidents of high arousal behaviours, 13.7% incidents of anxiety-related behaviours, and 3.5% incidents of aggressive behaviours.

In individual tests, the major behaviours that had the highest occurrences were friendly and fearful, whereas anxiousness, arousal and aggression had lower instances (Appendix D). However, there were higher instances of arousal in the toy interaction test which reflects the purpose of the test.

### 3.4. Relationships between Owner-Reported Dogs’ Behaviour in the Home and Behaviours Derived from the Formal Behaviour Assessment in the Shelter

All correlations were corrected using Bonferroni correction and varied in strength. Considering the overall behaviour assessment, there were positive Spearman rank correlations between the fear displayed in the assessment and the fear in non-social situations and stranger situations reported by the owner (Table 3). A friendly classification in the shelter assessment correlated negatively with stranger-directed fear reports by the owner. Aggression in the shelter correlated positively with touch sensitivity reports by the owner, both in the overall assessment and in the touch sensitivity test. In the latter test, friendliness correlated with the non-social fear reports by the owner.

In the Play interactions test in the shelter, fear correlated positively with stranger-directed and non-social fear and aggression in the home. Friendliness in this test correlated negatively with stranger-directed fear reports by the owner. In the Response to unusual/unpredictable stimuli test in the shelter, fear correlated positively with stranger-directed fear reports by the owner, which also correlated negatively with friendliness in the behaviour assessment. In the Food possession test in the shelter, friendliness correlated negatively with stranger-directed fear, and in the Toddler doll test, fear correlated positively with non-social fear reports by the owner, and aggression correlated with touch sensitivity reports by the owner.

### 3.5. Predictability of Behaviour Assessment

In the home environment, dogs whose owners reported low levels of stranger-directed fear had high levels of friendliness in the Overall shelter test and in the Response to Unusual/Unpredictable Stimulus, Food Possession, Stranger, and Toddler doll tests (Table 4). High levels of stranger-directed fear related positively to aggression in the Overall, Play interaction, Response to Unusual/Unpredictable Stimulus and Food Possession tests, to fearfulness in the Touch Sensitivity test and negatively to high arousal in the Toddler doll test. Owner-reported non-social fear and fear in the Exploration of room, Touch sensitivity and Response to unusual stimulus tests were related. Stranger-directed aggression reported by the owner was also related to fearfulness in the Touch sensitivity test. Owner-directed and reported aggression was negatively related to friendliness, fearfulness and high arousal in the Stranger test, and positively related to aggression in that test and the Toddler doll test. Familiar dog aggression reported by the owner was negatively related to friendliness, fearfulness and high arousal in the Toddler doll test and positively related to aggression in that test.

Touch sensitivity reported by the owner was negatively related with friendliness (Overall assessment, Response to unusual stimulus, Toddler doll, Time alone, Dog-to-dog interaction), high arousal (Overall assessment, Toddler doll, Touch sensitivity, Time alone), fearfulness (Touch sensitivity, Dog-to-dog interactions), and anxiety (Response to unusual stimulus, Toddler doll, Dog-to-dog interaction). There was a positive relationship between those related with aggression (Overall assessment, Touch sensitivity, Play interaction, Response to unusual stimulus, Toddler doll tests).

Attachment/attention seeking reported by the owner related negatively with friendliness (Response to unusual stimulus, Toddler doll), fearfulness (Overall assessment, Response to unusual stimulus, Toddler doll, Time alone), high arousal (Overall assessment, Play interaction, Response to unusual stimulus, Toddler doll), anxiety (Response to unusual stimulus, Toddler doll, Time alone). It related positively with aggression (Overall, Response to unusual stimulus, Toddler doll, Dog-to-dog interaction tests).

Excitability related negatively to fearfulness in Touch sensitivity, high arousal in Touch sensitivity, and it related positively to anxiousness in the Exploration of room, high arousal in the Exploration of room, and Time alone tests.

Energetic behaviour was related positively to high arousal in the Exploration of room, and aggression in Dog-to-dog interaction and negatively to friendliness in the Dog-to-dog interaction. Chasing was related negatively to anxiousness in the Toddler doll test.

## 4. Discussion

Behavioural assessments are used in the RSPCA Australian shelters to identify behavioural problems, determine suitability for adoption and to monitor the behaviour of each dog over time while in the shelter. The use of the behavioural assessment as a tool in combination with surrender information (home environment, in-home behaviour, and behaviour towards other dogs), veterinary history, in kennel observations, and staff feedback is thought to provide some representation of the dog’s behaviour. The behavioural assessment is not being used as a pass–fail tool, rather, it is used as one component of a toolbox to collect information over time. It is important to know how valid it is. The aim of this study was to determine if dogs’ home behaviour, measured using information provided by owners using the C-BARQ, was accurately reflected in the standardised RSPCA Queensland behaviour assessment. The study was conducted with dogs owned by members of the general public and therefore not dogs potentially negatively affected by stress due to time in the shelter.

Major themes identified in this study are consistent with the previous findings and results reported in previous studies, particularly in relation to fear, arousal, friendliness, and anxiousness [27,29]. The major tests that were most predictive of behaviour in a home environment were the exploration of room, touch sensitivity, and Response to unusual stimulus in regard to non-social fear. Stranger-directed fear was predictive in tests of touch sensitivity, and response to unusual stimulus response. Touch sensitivity was reflected in the corresponding test in the assessment. Owner-directed aggression was predicted in the stranger and toddler doll tests. Stranger-directed aggression was only identified in touch sensitivity in relation to fear. Excitability and energy were predicted in the exploration of room, touch sensitivity, and time alone tests. Finally, attachment was predicted in the tests related to the response to unusual stimulus, and toddler doll.

Overall friendliness identified during the play interactions, response to unusual stimulus, food possession, stranger, toddler doll and dog-to-dog interactions tests were reflected in the low scoring of the categories of energetic, fear and aggressive-related issues in C-BARQ. Categories of the C-BARQ that were not predicted in the tests were dog rivalry, dog-directed aggression, separation-related behaviours, trainability, and chasing.

There are few studies on the ability of an assessment to reflect previous home behaviour; rather, most literature looks at predicting future behaviour [8,13,14,25,31,32,33,34,35]. In this study, behaviour reported in the home showed a relationship with certain aspects of the behavioural assessment including fear, friendliness, anxiety, arousal and aggression.

The relationship between fear displayed in the assessment and owners’ indication of stranger-directed and non-social fear, aligns with previous findings of the predictability of fear [14,36]. In looking at C-BARQ categories, stranger-directed fear and aggression, and non-social fear in the home were related to fear observed in the exploration of room, touch sensitivity, and response to unusual stimulus. Non-social fear, stranger-directed fear, and aggression in the home were associated with increased odds of fearfulness in dogs in the assessment. This consistency of fear responses is to be expected, since the fear response is a manifestation of a survival response in the brain located in the amygdala, with the behavioural response created being very recognisable and easy to identify in all species [37]. Furthermore, the consistency of fear responses indicates a similarity of stimulus features and the demonstration of fearful behaviour requires appropriate environmental stimuli. One might expect to observe some consistency of fear responses in the home environment and shelter, even if people cannot categorise the motives/diagnosis of fear.

Mornement and co-authors [14] argued that general measures of anxiousness and fear measured in the Behaviour assessment for rehoming K9’s (B.A.R.K) protocol significantly predicted “Fearful/inappropriate toileting” behaviours post adoption. These results outline the stable predictiveness of fear consistent over a shelter to a post-adoption environment and therefore suggests the stability of fear over longitudinal periods. Foyer and co-authors [38] further reflected this in a study looking at behaviour in the first year of life and in a later temperament test in dogs. Results from the study outlined that dogs scoring high in categories of stranger-directed fear, non-social fear, and dog-directed fear showed a significantly lower rate of success 3 months later in the temperament test due to fear [38]. Therefore, it is of no surprise to observe consistency in the fear response seen in this study.

In relation to the friendliness displayed in the home environment and behaviour assessment, it is no surprise that it reflects previous findings [14]. Mornement and co-authors [14] found that post adoption, dogs greeting visitors in a friendly manner could be predicted by friendliness scores in B.A.R.K. However, it did not appear to be a reliable predictor of problem behaviours, such as overall aggression or destructive behaviour in shelters.

Furthermore, the predictability of behavioural problems outlined in the results using the owner information and the behaviour assessment could be due to the timing of the assessment. The assessment was conducted upon arrival, located in a room which was at a considerable distance from the main shelter. The stress of the shelter may cause the normal behavioural repertoire to change in the dog for the purpose of finding the best coping mechanism to deal with acute stress due to changes in the environment. Therefore, the timing of the assessment (currently at a minimum of 3 days after surrender) may cause the predictability of behaviour post adoption to be poorer due to the changes that stress can cause in normal behaviour. If we take human psychology as an example, humans that go into a novel environment which they have never been in before suffer an acute stress response. Humans, like all animals, need to adapt to a new environment; they can find positive and negative coping mechanisms to help with this which is then reflected in their behaviour [39]. If positive coping mechanisms are not found, then negative coping mechanisms are used, causing problem behaviours and sometimes addiction. Dogs that have never been in the novel environment before, such as the shelter, respond with an acute stress response due to social isolation from previous family, daily routine changes, disturbed feeding, walking, socialising, lack of handling and attachment figures, and sensory overstimulation. The dog must adjust to the new environment and if unable to cope effectively, behavioural problems start to occur. Once adopted, however, dogs then need to adjust back to home behaviour, which can be easy for most dogs but other dogs with behavioural problems may find this difficult. This is consistent with the findings of Mornement and co-authors [14] who indicated a high number of new adopters reporting signs of growling, snapping, and attempting to bite a person.

Not all instances of behaviour seen in the behavioural assessment-reflected responses to the C-BARQ questionnaire, including certain categories of aggression (dog-directed, stranger-directed), separation-related behaviours and possessive behaviours. Only one category of the C-BARQ, owner-directed aggression, showed consistency with the behaviour assessment stranger and toddler doll tests.

One might expect that stranger-directed aggression in these tests would be reported in the C-BARQ but this was not the case. A study by Dalla Villa et al. [25] outlined the use of the Socially acceptable behaviour (SAB) protocol for identifying categories of aggression. The results indicate that only categories of C-BARQ predictive of the SABS were associated with owner-reported aggression towards familiar people and familiar dogs, however, these were not directly measured by any of the SAB subtests. The identification of the category of aggression is difficult as there are numerous such categories [40] and aggression can be multifactorial. Therefore, this could explain the lack of results in the predictability of aggression towards another stimulus e.g., dog-directed and stranger directed. Without thorough examination of the context of aggression, the environment, and a comprehensive understanding of all factors at play, it is very difficult for assessments to correctly identify, let alone predict, categories of aggression.

Separation-related behaviours are difficult for assessments to identify predictably due to the multifactorial nature of the issue. The issue can be easily misclassified due to other underlying problems like attachment-seeking, general anxiety, fears, or phobias [41]. Furthermore, differential diagnosis should always be taken into account before outlining that the individual has separation anxiety. Storengen and co-authors’ [42] study of 215 dogs diagnosed with separation anxiety reported that only 18.5% of animals actually had only separation anxiety with no other behavioural problems, whereas 82.8% of the animals had other underlying behavioural problems in addition to separation anxiety, with the most common comorbidity being related to noise sensitivity (43.7%) [42].

Possessive behaviour has been reported in the literature to have a low predictability [13,14,31]. This may be due to the manifestation of the problem being environmentally based [13,31]. Possessive aggression is associated with a need to protect a resource from surrounding threats, however, once a threat is no longer present, the behaviour ceases, therefore it is not often seen in post-adoption environments. The study by Marder and co-authors [13] found that a little over half of the dogs with possessive behaviour in the shelter displayed these issues post adoption, whereas 22% of dogs identified in a shelter with no signs of possessive behaviours exhibited the behaviour post adoption. Furthermore, a study by Mohan-Gibbons [31] into the removal of the test, identified that there was a low risk of injury to handlers, volunteers, staff or adopters and no significant difference in the rate of returns. However, even though it was a low relative risk of occurrence in the home it is predictive, just not perfectly predictive. Possession aggression, however, can be stimulated by environmental or competition in the environment, therefore, if in a stable environment, such behaviours will decrease or cease. Therefore, in the current study, this could explain the low occurrence of possessive aggression, especially in the home environment.

Numerous possibilities exist that consider discrepancies between the behavioural assessment results and owner reports. A possibility is that the current standardised behaviour assessment may be adequate at identifying overall behaviours, however, unable to correctly identify certain behavioural problems. However, behavioural problems, such as dog-directed aggression or separation-related behaviours, may be inaccurately identified due to the misinterpretation of the behaviour by the owner in the home. For example, dogs that are reactive to other dogs at a distance could be misclassified as dog-aggressive or offensive aggressive, when what is being displayed is built-up frustration and hyperactivity towards other dogs. A study that assessed the behaviour of privately owned dogs using the Dutch socially acceptable behaviour test, found that a large portion of aggressive dogs remain undetected and the test was unsuitable for assessing types of aggression apart from fear [23]. The current results agree with this, outlining the high degree of detectability of fear.

There are limitations to this study. One limitation is that all dogs in this study had been in a home environment for over 6 months, and therefore, had an attachment figure. Attachment figures have previously been seen to have a significant impact on inhibitory control, problem-solving tasks and social interactions in comparison to dogs that were in shelters with no attachment figure [43,44,45]. Another limitation includes that the study population may not be representative of dogs that end up in shelters.

The results from this novel study suggest the benefit of an upon surrender assessment to increase the understanding of behaviour from the previous home environment. Early recognition of behavioural problems that include fear, anxiousness, arousal, and aggression can help dogs cope in the environment and allows behaviour modification to be implemented before the stressors of the shelters begin to have an effect [9].

## 5. Conclusions

This study suggested that the standardised behaviour assessment protocol used at an Australian shelter is a useful tool to reflect home behaviour when conducted upon entry to the shelter as mimicked in this study methodology, with friendliness, fearfulness, anxiousness, high arousal and certain categories of aggression measured by the C-BARQ being reflected in the assessment. The identification of behaviours of dogs upon entry can help to create a more comprehensive understanding of the dog’s behaviours in the home environment and further identify any behavioural issues/monitored throughout the stay in the shelter plus allow behaviour modification to start upon entry. Information can give a base line for the dogs before entry, thus allowing the longitudinal monitoring of behaviours and behavioural issues. Investigations into longitudinal monitoring from surrender to adoption, and the relationship of individual behavioural change over time, needs to be conducted.

## Figures and Tables

**Table 1:** Behaviours of dogs (n = 107) recorded for each body part, as well as the position in the room and movement types.

Mouth	Body	Tail	Tail Movement	Ears	Eyes	Position	Movement
Open/closed	Weight forward	Low	Wagging	Alert	Soft	Front	Pacing
Panting	Weight back	Med	Fast	Back	Hard	Bed	Sit/lay
Mouthing	Balanced	High	Stiff	Forward	Direct	Door	Stand
Lip lick	Relaxed	Tucked	Slow	Open	Squinting	Wall	Still
Snap	Tense		Loose		Whale eyes		
Bite	Lowered				Dilated		
Whining	Play bow				Targeted		
Barking	Jumping up				Diverted		
Growl	Lowered head						
Howling	Piloerect						
	Body curve						

**Table 2:** The behaviours contributing to the behavioural states fear, anxiety, aggression, arousal, and friendliness.

Fear															
Diverting	Ears back	Lip licking	Lowered body	Lowered head	Shiver	Stiff tail	Tail low	Tail tucked	Tense body posture	Weight back	Yawn				
Anxiety															
Fast tail	High tail	Jumping	Licking	Lip licking	Medium tail	Pacing	Panting	Stiff tail	Tense body	Weight back	Weight forward	Whining			
Aggression															
Biting	Ears forward	Growling	High tail	Lip licking	Lowered head	Medium tail	Snapping	Standing	Stiff tail	Still tail	Targeting	Vertical lip raise			
Arousal															
Barking	Diverting gaze	Fast tail	High tail	Jumping up	Jump off	Licking	Medium tail	Mouthing	Pacing	Panting	Weight forward	Whining			
Friendliness															
Balanced	Body curve	Direct eye	Ears forward	Ears open	Fast tail	Handler interaction	Jump	Medium tail	Play	Relaxed body	Slow tail	Sniff	Soft eye	Tail loose	Walking

**Table 3:** Significant (*p* < 0.01) Spearman rank correlations between the owner-reported dogs’ temperament/behaviour in the home and the behaviours derived from the formal behaviour assessment at the shelter.

Behaviour Assessment Test	Shelter Behaviours	Owner-Reported Temperament in the Home (C-BARQ)	Correlation Coefficient
Overall	Fear	Stranger-directed fear	0.34
		Non-social fear	0.36
	Friendliness	Stranger-directed fear	−0.32
	Aggression	Touch sensitivity	0.31
Touch sensitivity	Aggression	Touch sensitivity	0.27
	Friendliness	Non-social fear	−0.25
Play interactions	Fear	Stranger-directed fear	0.45
		Stranger-directed aggression	0.29
		Non-social fear	0.32
	Friendliness	Stranger-directed fear	−0.42
Response to Unusual/unpredictable stimulus	Fear	Stranger-directed fear	0.32
	Friendliness	Stranger-directed fear	−0.31
Food possession	Friendliness	Stranger-directed fear	−0.32
Toddler doll	Fear	Non-social fear	0.32
	Aggression	Touch sensitivity	0.32
			*p* < 0.01

**Table 4:** Significant (*p* < 0.01) relationships between the owner-reported temperament/behaviour and the behaviours derived from the overall behaviour assessment and individual tests, conducted in the shelter, determined by ordinal logistic regression.

Owner-Reported Temperament/Behaviour	Behaviour in Behaviour Assessment in Shelter	Coef.	Odds Ratio	Lower CI	Upper CI
	**Overall**				
Stranger-directed fear	Friendliness	0.20	1.22	1.07	1.41
	Aggression	−0.13	0.88	0.78	0.99
Touch sensitivity	Friendliness	0.16	1.17	1.03	1.33
	High arousal	0.12	1.13	0.99	1.30
	Aggression	−0.14	0.87	0.77	0.98
Attachment/attention-seeking	Fearfulness	0.13	1.14	1.01	1.30
	High arousal	0.17	1.19	1.03	1.36
	Aggression	−0.13	0.88	0.78	0.99
	**Exploration of room**				
Non-social fear	Fearfulness	−0.04	0.96	0.93	0.99
Excitability	Anxiousness	−0.06	0.94	0.89	1.00
	High arousal	−0.05	0.95	0.91	0.99
Energetic	High arousal	−0.04	0.96	0.92	1.00
	**Touch sensitivity**				
Stranger-directed fear	Fearfulness	−0.04	0.96	0.93	0.99
Non-social fear	Fearfulness	−0.03	0.97	0.94	0.99
Stranger-directed aggression	Fearfulness	−0.04	0.96	0.93	0.99
Touch sensitivity	Fearfulness	0.15	1.16	1.03	1.30
	Anxiousness	0.17	1.18	1.03	1.35
	High arousal	0.15	1.16	1.02	1.32
		−0.10	0.91	0.83	0.99
Excitability	Fearfulness	0.15	1.16	1.03	1.30
	High arousal	0.15	1.17	1.02	1.33
	Aggression	0.15	1.17	1.02	1.33
	**Play interactions**				
Stranger-directed fear	Friendliness	0.15	1.16	1.05	1.27
	Aggression	−0.12	0.88	0.81	0.97
Touch sensitivity	Aggression	−0.12	0.89	0.81	0.97
Attachment/attention-seeking	High arousal	0.12	1.13	1.02	1.25
	**Response to unusual/unpredictable stimulus**				
Stranger-directed fear	Friendliness	0.13	1.13	1.04	1.24
	Fearfulness	−0.04	0.96	0.94	0.99
	Aggression	−0.09	0.91	0.84	0.99
Non-social fear	Fearfulness	−0.03	0.97	0.95	1.00
Separation related behaviours	Aggression	−0.08	0.92	0.85	1.00
	Friendliness	0.09	1.09	1.01	1.19
Attachment/attention-seeking	Friendliness	0.15	1.16	1.05	1.29
	Fearfulness	0.10	1.10	1.02	1.20
	Anxiousness	0.12	1.13	1.03	1.23
	High arousal	0.11	1.12	1.02	1.23
	Aggression	−0.09	0.91	0.84	0.99
Touch sensitivity	Friendliness	0.10	1.11	1.02	1.20
	Anxiousness	0.13	1.14	1.02	1.27
	Aggression	−0.09	0.91	0.84	0.99
	**Food possession**				
Stranger-directed fear	Friendliness	0.13	1.14	1.02	1.28
	Aggression	−0.11	0.89	0.80	0.99
	**Stranger**				
Stranger-directed fear	Friendliness	0.10	1.10	1.01	1.21
Owner-directed aggression	Friendliness	0.12	1.13	1.02	1.25
	Fearfulness	0.12	1.12	1.02	1.24
	High arousal	0.13	1.13	1.01	1.27
	Aggression	−0.13	0.88	0.80	0.97
	**Toddler doll**				
Stranger-directed fear	High arousal	0.12	1.13	1.01	1.26
	Friendliness	0.09	1.10	1.00	1.20
Familiar dog aggression	Friendliness	0.12	1.13	1.03	1.24
	Fearfulness	0.11	1.11	1.01	1.22
	High arousal	0.13	1.14	1.03	1.28
	Aggression	−0.12	0.89	0.81	0.98
Owner-directed aggression	Aggression	−0.13	0.88	0.79	0.97
Attachment/attention-seeking	Friendliness	0.11	1.11	1.02	1.21
	Fearfulness	0.12	1.13	1.04	1.24
	Anxiousness	0.17	1.19	1.08	1.32
	High arousal	0.16	1.18	1.07	1.29
	Aggression	−0.12	0.89	0.82	0.97
Touch sensitivity	Friendliness	0.11	1.12	1.03	1.22
	Anxiousness	0.11	1.12	1.01	1.24
	High arousal	0.10	1.10	1.01	1.21
	Aggression	−0.11	0.90	0.83	0.97
Chasing	Anxiousness	0.11	1.11	1.01	1.23
	**Time alone**				
Attachment/attention-seeking	Fearfulness	0.11	1.12	1.01	1.24
	Anxiousness	0.15	1.17	1.04	1.31
Touch sensitivity	Friendliness	0.11	1.12	1.01	1.24
	High arousal	0.14	1.15	1.02	1.29
Excitability	High arousal	−0.04	0.96	0.92	1.00
	**Dog-to-dog interaction**				
Attachment/attention-seeking	Aggression	−0.08	0.93	0.86	1.00
Touch sensitivity	Friendliness	0.09	1.10	1.01	1.19
	Anxiousness	0.13	1.14	1.01	1.29
Energetic	Friendliness	0.09	1.10	1.01	1.20
	Aggression	−0.09	0.92	0.85	0.98

## Appendix A. RSPCA Standardised Behavioural Assessment

### Appendix A.1. Test 1: Exploration of Room

#### Appendix A.1.1. Exploring the Room

The assessor entered the room, dropped the lead attached to the dog, and sat in the centre on a chair. Then, the observer started a timer and waited for 1 min without any interaction with the dog by either person.

#### Appendix A.1.2. Sociability to Assessor

At the end of exploring the room, the assessor called the dog to them in a friendly voice, remaining in the chair with no other body movement. If there was no response, a second attempt was made, and if still no response the assessor clapped their hands on their lap and said ‘come here’ in the direction of the dog, trying at least three times to call the dog to them. When the dog came (at the first, second, or third call), the assessor picked up the leash and then stroked the dog from the base of the neck to the tail three times. If the dog did not respond to the first, second, or third call, the assessor approached the dog, picked up the leash, and gave the dog three strokes from the base of the neck to the tail. Following each stroke, the observer and assessor counted 10 s, with the behaviours exhibited noted.

### Appendix A.2. Test 2: Tolerance to Handling

There were three components to the test, namely touch sensitivity to collar, stroke, and feet. The assessor dropped the leash and held the dog’s collar. After 3 s, the handler stroked the dog from head to tail. With the dog standing, the other assessor (in the standing position, or crouching if a small breed of dog) picked up the dog’s rear inside foot, then the front inside foot, then reached over its back to pick up its rear outside foot, and finally the front outside foot. Each foot was held for 2 s. After picking up all four paws in this manner, the assessor stood for 10 s with no dog interaction and finally removed the dog’s leash.

### Appendix A.3. Test 3: Startle Response

There were two components: startle response and recovery to stimulus. At the end of Test 2, the assessor created a loud sound using a book on a bench or a desk (startle response). The assessors recorded recovery.

### Appendix A.4. Test 4: Toy Interactions

Three toys were used in this testing procedure: tennis ball, squeaky toy, and tugging rope. A tennis ball was shown to the dog and gently thrown across the room, and the assessor verbally engaged the dog in play. If the dog picked up the ball, the assessor waited to see if it returned to the assessor without encouragement. If it did not, the assessor encouraged the dog to bring the ball back by calling his/her name and saying “come”. If the dog still did not return, the assessor went to the dog.

In both situations, the assessor waited 10 s to see if the dog dropped the ball. If it did not, they asked the dog to “drop it”. If the dog did not respond, then a second command was given, “give”, and if necessary, a third attempt, “out”, was tried. If the dog did not respond to these commands, the assessor approached the dog carefully and removed the ball from the dog’s mouth. These steps were repeated for a second throw and after completion, the assessor waited 10 s with no interaction before moving on to the next toy, the squeaky toy, and after that, the tugging rope. The same sequence was used for each toy. After completing all three toys, the assessor moved on to the next test.

### Appendix A.5. Test 5: Response to Unusual/Unpredictable Stimulus

The assessor gently moved the dog to the opposite end of the room and left it standing against the wall. Then, they gently moved one hand over its head, down toward the back to gently tap the rump area, and then ran across the room, laughing and waving arms, followed by suddenly stopping, folding their arms, and ignoring the dog. The tap, run, and freeze series was repeated a second time. The assessor waited for 10 s after the run and freeze, ignoring the dog, before moving onto the next test. The dog was then placed back on the leash.

### Appendix A.6. Test 6: Resource Guarding

There were four components to the test: wet food, dry kibble/biscuits, pig’s ear and bone. The assessor tethered the dog to the wall for safety reasons, and proceeded to show the dog wet canned food, smeared in a bowl. The bowl was then placed near the dog at the end of the leash perimeter, allowing the dog to begin eating for 2 s. The assessor then proceeded with a plastic hand, walking to the side of the dog while it was eating. Using the fake hand, the assessor patted the dog on the head, continuing to stroke down its back and body twice. The fake hand was then placed 5 cm in front of the bowl and moved around in a semi-circle. The hand was then placed on the inside edge of the bowl and moved around the edge of the bowl next to the dog’s face, without touching it. Finally, the bowl was pulled away from the dog using the fake hand. The bowl was then returned to the dog, which was observed for 10 s.

The assessor then gave the dog a pig’s ear or bone, depending on the dog’s food interest, and it was allowed to chew it for 30 s. The steps above with wet food were repeated; then, the assessor attempted to retrieve the food, asking the dog to “drop it”, “leave it”, or “give” before attempting to retrieve it by offering a new food that is novel.

### Appendix A.7. Test 7: Stranger Interaction

There were three components to the test: the entry, approach and exit of a stranger. The assessor placed the dog on a leash as the observer exited the room and returned dressed in a reflective vest, large brimmed hat and using a walking stick. The observer entered the room, and bent down to extend an open flat hand as if to pat the dog on the head. The observer then talked to the dog normally and stopped for 3 s, allowing the dog to approach. If the dog approached, the observer patted the dog on the top of its head for 3 s. If the dog did not approach, it was observed for 10 s, with an emphasis on any interaction between the assessor and/or the observer.

### Appendix A.8. Test 8: Fake Toddler Interaction

There were two components of the test: the approach of the toddler doll and the exit/removal of the toddler doll. The assessor stood and held the dog’s leash while the observer exited the area and returned carrying a toddler doll simulating a small child. Once the toddler was within the leash perimeter from the dog, the observer placed the doll on the floor facing the dog, with the doll’s arm extended toward the dog. The assessor allowed the dog to approach if it desired. If the dog did not approach the observer, it was observed for 20 s. After this, the assessor picked up the toddler doll and walked back out of the room. The assessor allowed the dog to follow to the door or move away from stimulus.

### Appendix A.9. Test 9: Fake Cat

The assessor stood and held the dog’s leash while the observer exited the area and returned carrying a fake cat as if it were a “real” cat. Once the fake cake was within the leash perimeter from the dog, the observer placed the fake cate on the floor facing the dog. The assessor allowed the dog to approach if he/she wanted to. However, if the dog did not approach the observer, the dog was observed for 20 s with the fake cat present.

### Appendix A.10. Test 10: Time Alone

The assessor and observer removed the leash from the dog and left the room for 2 min, with a video camera in the front of the room monitoring behaviour and vocalisations. Then, the assessor and observer re-entered through the same door.

### Appendix A.11. Test 11: Behaviour with Another Dog

There were three components to the test: walking parellel, circling activity, and nose-to-nose interaction. This test was conducted in a yard (10−20 m), allowing adequate space between the test dog and another dog. Each dog had an assessor, who interacted with their dog by giving treats and ignoring the other assessor and dog. The assessor had a short, 1 m leash, so that the dog walked close to the assessor. At the start, both assessors walked parallel to each other, 5 m apart, with the dogs on the outside. If one or both dogs were reactive and pulled toward each other, the distance between the assessors was increased. If both dogs were relaxed and focused on their assessor, the assessors moved the dogs to an exercise circle. If the dogs did not breach a minimum distance of 5 m between them, they were introduced on opposite sides of a fence. Then followed a circling activity, which required one assessor to stand still with their dog on no more than 1.5 m of leash while the other assessor and their dog completed a circle around the assessor. The assessors then swapped places and repeated the circling activity. If no adverse behaviours were displayed, the assessor in the middle of the circle remained at that location, ensuring that the only tension on the leash was from the dog. The other assessor identified the leash threshold of the dog in the centre and moved close enough to allow the dogs to be nose to nose, also ensuring that the only tension on their leads was caused by the dog pulling, not them pulling against the dog. Once the leads became loose, and the dogs stopped pulling against the assessor, the assessors took a step closer to each other, allowing the dogs to interact if they chose. Leashes remained loose. If there were signs of adverse reactions or aggression, the dogs were separated by increasing the threshold.

## Appendix B

**Table A1:** C-BARQ Categories and Descriptions.

C-BARQ Categories	Description	
Stranger-directed aggression	Dog acts aggressively	When approached directly by an unfamiliar male adult while being walked or exercised on a leash
		When approached directly by an unfamiliar female adult while being walked or exercised on a leash
		When approached directly by an unfamiliar child while being walked or exercised on a leash
		Toward unfamiliar persons approaching the dog while it is in the owner’s car
		When an unfamiliar person approaches the owner or a member of the owner’s family at home
		When an unfamiliar person approaches the owner or a member of the owner’s family away from home
		When mailmen or other delivery workers approach the home
		When strangers walk past the home while the dog is in the yard
		When joggers, cyclists, roller skaters, or skateboarders pass the home while the dog is in the yard
		Toward unfamiliar persons visiting the home
Owner-directed aggression	Dog acts aggressively	When verbally corrected or punished by a member of the household
		When toys, bones, or other objects are taken away by a member of the household
		When bathed or groomed by a member of the household
		When approached directly by a member of the household while it is eating
		When food is taken away by a member of the household
		When stared at directly by a member of the household
		When stepped over by a member of the household
		When a member of the household retrieves food or objects stolen by the dog
Stranger-directed fear	Dog acts anxious or fearful	When approached directly by an unfamiliar male adult while away from the home
		When approached directly by an unfamiliar female adult while away from the home
		When approached directly by an unfamiliar child while away from the home
		When unfamiliar persons visit the home
Non social fear	Dog acts anxious or fearful	In response to sudden or loud noises
		In heavy traffic
		In response to strange or unfamiliar objects on or near the sidewalk
		During thunderstorms firework displays, or similar
		When first exposed to unfamiliar situations
		In response to wind or wind-blown objects
		Towards another (familiar) dog in your household.
		When approached at a favorite resting/sleeping place by another household dog
Dog Rivalry	Dog acts aggressively	When approached while eating by another household dog
		When approached while playing with/chewing a favorite toy, bone, object by another household dog
Dog-directed aggression	Dog acts aggressively	When approached directly by an unfamiliar male dog while being walked or exercised on a leash
		When approached directly by an unfamiliar female dog while being walked or exercised on a leash
		Toward unfamiliar dogs visiting the home
		When barked, growled or lunged at by an unfamiliar dog
Dog-directed fear	Dog acts anxious or fearful	When unfamiliar dogs visit the home
		When barked, growled or lunged at by an unfamiliar dog
		When approached directly by an unfamiliar dog of the same or larger size
		When approached directly by an unfamiliar dog of a smaller size
Separation-related behavior	Dog displays	Shaking, shivering, or trembling when left or about to be left on its own
		Excessive salivation when left or about to be left on its own
		Restlessness, agitation, or pacing when left or about to be left on its own
		Whining when left or about to be left on its own
		Barking when left or about to be left on its own
		Howling when left or about to be left on its own
		Chewing or scratching at doors, floor, windows, and curtains when left or about to be left on its own
		Loss of appetite when left or about to be left on its own
Attachment or attention-seeking behavior	Dog	Displays a strong attachment for a particular member of the household
		Tends to follow a member of household from room to room about the house.
		Tends to sit close to or in contact with a member of the household when that individual is sitting down
		Tends to nudge, nuzzle, or paw a member of the household for attention when that individual is sitting down
		Becomes agitated when a member of the household shows affection for another person
		Becomes agitated when a member of the household shows affection for another dog or animal
Trainability	Dog	Returns immediately when called while off leash
		Obeys a sit command immediately
		Obeys a stay command immediately
		Will fetch or attempt to fetch sticks, balls, and other objects
		Seems to attend to or listen closely to everything the owner says or does
		Is slow to respond to correction or punishment
		Is slow to learn new tricks or tasks
		Is easily distracted by interesting sights, sounds, or smells
Chasing	Dog	Acts aggressively toward cats, squirrels, and other animals entering its yard
		Chases cats if given the chance
		Chases birds if given the chance
		Chases squirrels and other small animals if given the chance
Excitability	Dog overreacts or is excitable	When a member of the household returns home after a brief absence
		When playing with a member of the household
		When the doorbell rings
		Just before being taken for a walk
		Just before being taken on a car trip
		When visitors arrive at its home
Touch sensitivity	Dog acts anxious or fearful	When examined or treated by a veterinarian
		When having its claws clipped by a household member
		When having feet toweled by a household member
		When groomed or bathed by a household member
Energy	Dog	Dog is playful, puppyish, and boisterous
		Dog is active, energetic, and always on the go

## Appendix C

**Table A2:** Number (and %) of respondents (n:107) classifying their dogs in each of five levels on a scale of increasing intensity of behaviour exhibited at home, using the C-BARQ Categories.

Behaviour	Target of Behaviour	Scale ^†^									
		0		1		2		3		4	
Fear	Stranger-direct	73	(68.2)	25	(23.4)	5	(4.67)	2	(1.86)	2	(1.86)
	Non Social	60	(56.1)	33	(30.8)	12	(11.2)	1	(0.93)	1	(0.93)
	Dog directed	49	(45.8)	36	(33.6)	13	(12.1)	8	(7.47)	1	(0.93)
Aggression	Stranger-directed	77	(72.0)	24	(22.4)	5	(4.67)	1	(0.93)	0	(0.00)
	Owner-directed	101	(94.4)	2	(1.87)	4	(3.73)	0	(0.00)	0	(0.00)
	Dog directed	36	(33.6)	25	(23.0)	27	(25.2)	11	(10.3)	2	(1.86)
	Familiar dog	71	(66.3)	24	(22.4)	8	(7.47)	4	(3.73)	0	(0.00)
Separation related problems		82	(76.6)	21	(19.6)	3	(2.80)	1	(0.93)	0	(0.00)
Attention-seeking		1	(0.93)	33	(30.8)	52	(48.6)	18	(16.8)	2	(1.86)
Touch sensitivity		60	(56.1)	33	(30.8)	12	(11.2)	1	(0.93)	1	(0.93)
Chasing behaviour		27	(25.2)	16	(15.0)	28	(26.2)	32	(29.9)	4	(3.73)
Excitability		1	(0.93)	33	(30.8)	46	(43.0)	23	(21.5)	4	(3.73)
Energetic		9	(8.41)	32	(29.9)	45	(42.1)	17	(15.9)	4	(3.73)
Trainability		1	(0.93)	7	(6.54)	68	(63.6)	31	(29.0)	0	(0.00)

^†^ Fear, 0 no fear or anxiety—4 extreme fear, both stranger, non-social and dog fear; aggression, 0 none—4 serious, separately scored for stranger-, owner-, dog and familiar dog-directed; separation-related problems, from 0 never—4 always; attachment/attention-seeking, from 0 never—4 always; touch sensitivity, from 0 never—4 always; excitability, from 0 calm to 4 extremely excitable; chasing, energy, and trainability, from 0 never—4 always.

## Appendix D

**Table A3:** Percentage of coded durations and frequencies of the five behavioural categories (friendliness, fear, anxiety, arousal and aggression) during each subtest in the standardised behaviour assessment.

Test	Friendliness		Fear		Anxiety		Arousal		Aggression	
	*F*	*D*	*F*	*D*	*F*	*D*	*F*	*D*	*F*	*D*
Exploration	30.6	38.5	19.8	32.5	24.8	15.7	21.0	11.8	3.8	1.5
Tolerance to Handling	31.8	37.5	30.7	39.4	19.1	13.8	9.6	6.8	8.9	2.5
Toy interaction	46.6	44.3	16.3	18.8	16.3	14.8	16.9	19.9	3.9	2.3
Response to stimulus	35.2	37.1	22.3	27.4	20.5	16.9	18.2	15.9	3.8	2.7
Resource guarding	41.0	45.6	26.1	30.3	15.7	11.0	12.9	11.7	4.3	1.5
Stranger	37.0	40.9	25.0	27.1	16.4	13.6	15.4	15.4	6.1	3.0
Toddler doll	38.2	40.8	25.8	27.1	14.4	13.0	14.4	15.3	7.2	3.8
Time alone	26.3	39.3	13.8	29.6	28.8	16.6	28.6	12.5	2.4	2.0
Dog to Dog	35.5	47.2	21.2	25.1	19.2	12.6	17.4	11.5	6.6	3.5
